# Low dose radiation 18F–fluoride PET/CT in the assessment of Unilateral Condylar Hyperplasia of the mandible: preliminary results of a single centre experience

**DOI:** 10.1186/s41824-018-0025-3

**Published:** 2018-04-09

**Authors:** G. M. Lima, S. Diodato, E. Costabile, G. Cicoria, S. Civollani, C. Marchetti, P. L. Guidalotti, C. Pettinato, C. Nanni, S. Fanti

**Affiliations:** 1 Nuclear Medicine Department, S.Orsola-Malpighi Hospital, University of Bologna, Bologna, Italy; 2 Maxillo-Facial Surgeon Department, S.Orsola-Malpighi Hospital, University of Bologna, Bologna, Italy; 3 Department of Medical Physics, S.Orsola-Malpighi Hospital, University of Bologna, Bologna, Italy

**Keywords:** Fluoride PET/CT, 18F–NaF, UCH, Unilateral condylar hyperplasia, Hypercondylia

## Abstract

**Background:**

Unilateral condylar hyperplasia (UCH) of the mandible, or Hypercondylia, is a pathological condition that determines an abnormal growth of the affected condyle.

Bone SPECT with Tc99m-diphosphonates is a successful tool in the diagnosis of UCH. EANM guidelines also suggest the use of 18F–NaF PET/CT, though it leads to a higher radiation exposure.

**Aim:**

As UCH patients are young, we aimed to develop a low dose 18F–Fluoride PET/CT protocol and compare it to a standard injected activity scan, to assess if the image quality remains unchanged.

**Materials and methods:**

We prospectively enrolled 20 patients (7 males, 13 females, mean age 23.2) with UCH, who underwent 18F–NaF PET/CT to assess the hypercondylia. We administered a low activity of 18F–NaF (2.9 MBq/kg) in 15 patients and a standard activity (5.3 MBq/kg) in 5 patients. Activity range was chosen according to 2015 EANM guidelines.

To determine if the scans with low radiotracer activity were “diagnostic” such as those with standard activity, two expert nuclear medicine physicians, unaware of the administered activity, independently reviewed the scans and expressed a final qualitative judgment in terms of “diagnostic”/“non-diagnostic” scan. Furthermore, we compared the effective dose of a low injected activity PET/CT to the standard one and to a Bone SPECT performed with standard injected activity of Tc99m-diphosphonates.

**Results:**

Reviewers classified 19 of 20 scans as “diagnostic”. Only one of them was classified as “non diagnostic” due to condylar arthrosis that disturbed the correct evaluation of condylar radiotracer uptake. The effective dose of a 18F–Fluoride PET/CT, in patient of 70 kg, is about 3.5 mSv in scans performed with 2.9 MBq/kg [0.017 mSv/MBq × 2.9 MBq/kg × 70 kg] and about 6.3 mSv in ones performed with 5.3 MBq/kg [0.017 mSv/MBq × 5.3 MBq/kg × 70 kg]. The effective dose of 99mTc-MDP bone SPECT is about 3.2 mSv [0.0043 mSv/MBq × 740 MBq of 99mTc-MDP].

**Discussion:**

18F–NaF PET/CT performed with a low radiotracer activity allows a good assessment of UCH similar to that performed with an ordinary activity. The effective radiation dose of a low-injected activity PET/CT is significantly lower than an ordinary-injected activity and is not significantly higher than the most used Bone SPECT. Moreover PET/CT is performed in 1.5 h while Bone SPECT requires at least 3.5 h.

**Conclusions:**

The 18F–Fluoride PET/CT procedure could be performed with 2.9 MBq/Kg (minimum 185 MBq, recommended at least 200 MBq) of 18F–NaF to minimize the effective radiation dose received, maintaining the quality of the scan. Further studies including a larger number of patients and clinical follow-up are needed to confirm our preliminary findings.

## Background

Unilateral condylar hyperplasia (UCH) of the mandible, or Hypercondylia, is a rare pathological condition, in which an abnormal growth of the condyle causes facial asymmetry (Cervelli et al., 2008; Ahmed et al., 2016). It affects young people at pre-puberty or puberty (both sexes may be affected but prevalence is higher in females) (Raijmakers et al., 2012).

Obwegeser and Makek, in 1986, distinguished three different facial malformations due to condylar hyperplasia: hemimandibular hyperplasia (HH), hemimandibular elongation (HE) and a mixed form (Obwegeser & Makek, 1986).

HH is characterized by a unilateral three-dimensional enlargement of mandible (in both horizontal and vertical dimensions) with shifting of the midpoint of the chin to the unaffected side (Mohan et al., 2013).

HE is characterized by a unilateral horizontal elongation of mandible and the rotation of the chin is not so prominent (Mohan et al., 2013).

UCH, less frequent, may cause also functional alterations of the temporo-mandibular joint such as obstruction in mouth opening (sometimes associated with wobbling of the occlusion plane), third class occlusion, contralateral crossed bite, pain (especially during the palpation) and joint noise (Cervelli et al., 2008; Ferreira et al., 2014).

Pathogenesis of UCH is unclear (Olate et al., 2013). However, it seems that many factors could induce a unilateral abnormal growth: circulatory default, traumas, hormonal dysfunctions, cartilage exostosis, infections and arthrosis (Cervelli et al., 2008; Gn et al., 2015; Wolford et al., 2002).

From the functional point of view, many authors distinguish UCH in two forms: active hypercondylia (if the mandibular condyle is still in active growth) and inactive hypercondylia (if the growth is completed) (Pripatnanont et al., 2005). These different forms may require different treatments. In fact, many authors agree that an inactive UCH should be treated with a Le Fort I osteotomy while an active UCH should be treated with a Le Fort I osteotomy + condylectomy (Ferreira et al., 2014; Olate et al., 2013; Marchetti et al., 2000).

The diagnostic process of UCH includes a clinical approach, supported by imaging, both anatomical and functional.

In order to confirm the clinical suspicion, patients with facial asymmetry usually perform a radiographic examination. However, to date, three-dimensional imaging procedures are preferred: the dental Cone Beam Computed Tomography (CBCT) seems to be the best radiological three-dimensional procedure to evaluate the morphological condition of the condyle (Nolte et al., 2016).

After that, a functional nuclear medicine imaging procedure is required to distinguish between active UCH (affected mandibular condyle still in a phase of active growth) and inactive UCH (no signs of active growth in affected mandibular condyle).

This distinction between active/inactive hypercondylia is critical for choosing the appropriate surgical treatment (Pripatnanont et al., 2005). Many studies in literature indicate Bone SPECT (99mTc-diphosphonates, Single Photon Emission Computed Tomography) as a successful technique to distinguish between active and inactive growth. Because the diphosphonates uptake depends on the osteoblastic activity, active centres of ossification are detected as areas of increased uptake of the radiotracer (Wen et al., 2014; Saridin et al., 2011).

The 2015 EANM guidelines suggest also the use of 18F–NaF PET-CT (18F–Fluoride PET/CT), in the assessment of UCH (Beheshti et al., 2015): in fact, incorporation of 18F–fluoride ions into the bone matrix is higher in sites of new mineralization such as during bone growth. Compared to Bone SPECT, 18F–NaF PET-CT provides better spatial resolution images; furthermore, the protein bound fraction of 18F–NaF is lower than the 99mTc-MDP and the clearance from the blood is faster, resulting in lower background radiotracer signal (Laverick et al., 2009). Drawbacks of Fluoride PET/CT include the dose delivered to the patient. According to EANM guidelines, 5.3 MBq/Kg of 18F–NaF (on average) should be administered, providing a dose higher than bone SPECT.

## Aim

The aim of this study was to develop a low dose 18F–NaF PET/CT protocol and compare it to a standard injected activity scan, to assess if the image quality remains unchanged.

In standard 18F–NaF PET/CT protocol the injected activity is about 5.3 MBq/Kg of 18F–NaF.

In our optimized protocol we decided to inject the lowest activity allowed by 2015 EANM guidelines that is 2.9 MBq/Kg of 18F–NaF (minimum 185 MBq).

## Methods

From May 2015 to July 2017, we prospectively enrolled 20 patients (7 males, 13 females, mean age 23.2, range 15–43 years) with noticeable facial-mandibular asymmetry strongly suspicious for UCH. All patients were clinically examined by two maxillo-facial surgeons experts in UCH; the initial situation was accurately documented with iconographic material and stored in to the patient clinical history.

**Fig. 1:**
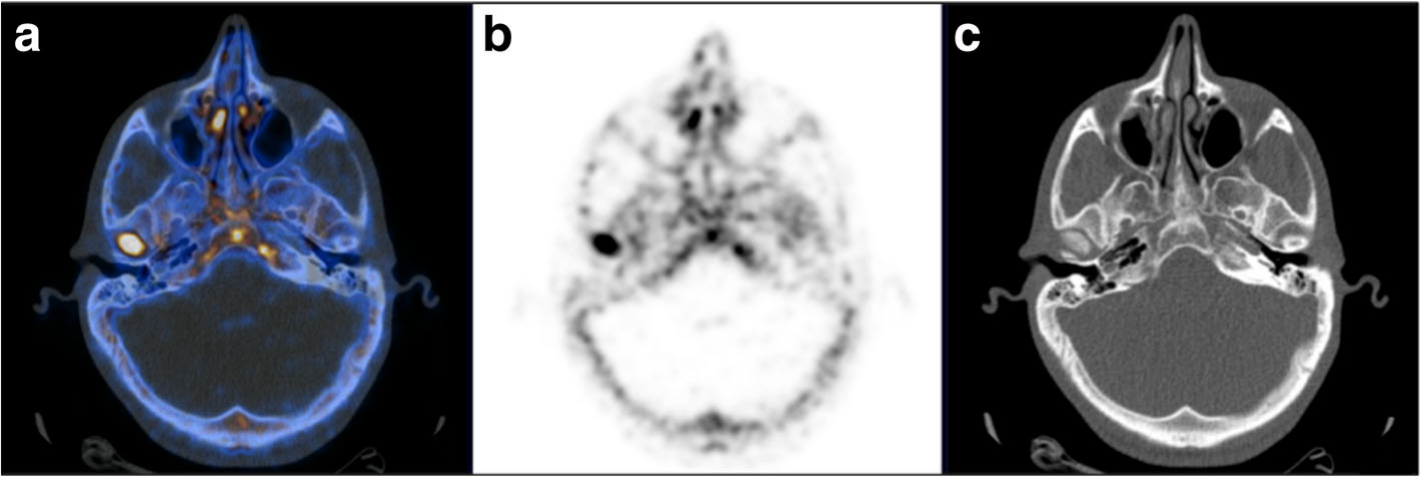
**a** axial 18F–fluoride PET/CT fusion scan of a patient affected by UCH in active growth: radiotracer uptake on right mandibular condyle; **b** axial 18F–fluoride PET scan only; **c** axial CT scan only

**Fig. 2:**
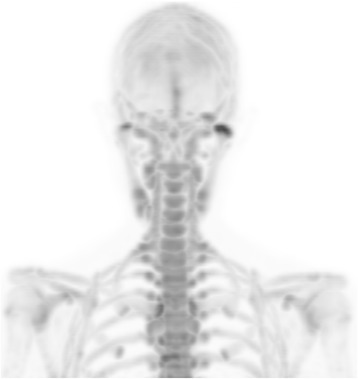
18F–Fluoride PET/CT MIP reconstruction

All patients underwent a 18F–NaF PET/CT (Fig. 1a) to confirm the presence of condylar alteration (CT images, Fig. 1c) and distinguish between active hypercondylia and inactive hypercondylia (PET images, Figs. 1b, 2).

Before the examination, women in childbearing age signed an informed consent to exclude pregnancy. Patients were asked to drink water (at least 50 cl) after radiotracer injection. No other cares were required. Patients were divided into two groups. Group A (5 patients) were injected with the standard activity of 5.3 MBq/Kg of 18F–NaF while patients of group B (15 patients) were injected with the lowest activity allowed by 2015 EANM guidelines (Beheshti et al., 2015) that is 2.9 MBq/Kg of 18F–NaF (minimum 185 MBq).

All patients underwent the PET/CT scan 1 h after injection of 18F–NaF using a Discovery 710 TF PET/CT system (GE Medical System, Waukesha, WI).

**Fig. 3:**
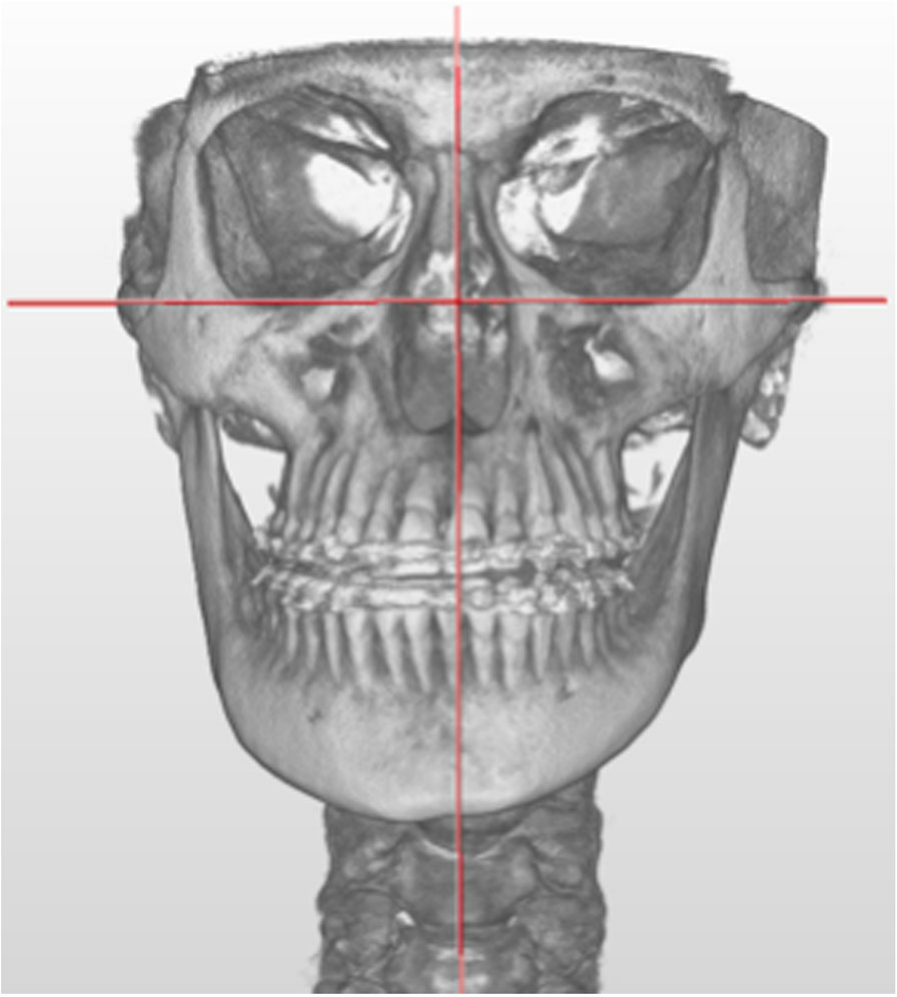
3D CT-reconstruction performed using CT data of PET/CT scan: specific software allows a computer-assisted three-dimensional surgical planning; patient with UCH

The first step of the acquisition protocol was a low dose CT performed from the glabella to the chin. CT acquisition parameters were: 120 kV, 10 mA, helical acquisition (0.8 s/rot), 3.7 mm slice thickness (reconstruction 1.25 mm) and matrix 512 × 512. A 3D reconstruction software was used to plan the surgery (Fig. 3).

The second step was a single-bed 10 min Time of flight PET scan, performed from the glabella to the chin. PET images were reconstructed using a View Point HD iterative reconstruction algorithm using both time of flight and point spread function recovery resolution information.

The radiotracer uptake of each mandibular condyle was measured by two expert physicians (GML, SD) as SUVmax (Max Standardized Uptake Value of the radiotracer) in the VOI (Volume-of-Interest) positioned on each condyle. Then the percentage difference of the radiotracer uptake between the two condyles was also calculated using the following formula:

$$ SUV\mathit{\max}\% Difference=\left(\frac{SUV_{\mathit{\max}- ac}-{SUV}_{\mathit{\max}- nac}}{SUV_{\mathit{\max}- ac}+{SUV}_{\mathit{\max}- nac}}\right)\ast 100 $$

Where SUV_max-ac_ is the SUV max of the affected condyle and SUV_max-nac_ is the SUV max of the non-affected condyle.

If the higher radiotracer uptake is on cortical bone this could be due to arthrosis. In this case, SUV measurement and UCH assessment are not optimal.

Based on literature (Ahmed et al., 2016), a SUVmax percentage difference > 10% was considered suspected for an abnormal unilateral active condylar growth and patients were considered for a mandibular condylectomy of the hotter side (in addition to the Le Fort I). However, a SUVmax percentage difference between 10 and 15% was classified as “grey zone”.

The CT images of the PET/CT scan were used for a precise surgery planning.

Patients with a SUVmax percentage difference < 10% were classified as suspected for inactive UCH and candidates to a conservative treatment of the condyle (only Le Fort I osteotomy).

To determine if the PET/CT scans performed with a low dose of radiotracer (group B) were effectively “diagnostic” such as those performed with a standard dose of radiotracer (group A), we asked to two expert nuclear medicine physicians (CN, PLG), unaware of the administered activity, to review the scans and express a final qualitative judgment in terms of “diagnostic or non-diagnostic scan”.

In terms of effective dose, data were derived from ICRP recommendations (ICRP 128): in particular effective dose from 99mTc- diphosphonates is 0.0043 mSv/MBq, while effective dose for 18-F Fluoride is 0.017 mSv/MBq (Mattsson et al., 2015). CT doses were derived from dose reports stored on the system for each acquisition. We compared the effective dose of a PET/CT performed with a low injected activity to a PET/CT performed with a standard injected activity and to a Bone SPECT performed with a standard injected activity of 99mTc-diphosphonates (bone SPECT, to date, is the most used nuclear medicine imaging procedure in UCH assessment).

## Results

Nineteen out of 20 examinations were classified as “diagnostic” by reviewers. Only one of them was classified as “non diagnostic” because of an augmented uptake area in the bone cortical medial side of the non-affected condyle, probably due to arthrosis, that disturbed the correct process of SUVmax measurement in this patient.

Low dose PET/CT scans resulted of good quality as well as ordinary dose scans.

Low dose scans performed with at least 200 MBq of 18F–NaF resulted in a better examination quality compared to ones performed with less than 200 MBq.

For all patients, the suspicious side, the effective increased uptake side, the SUVmax measured both in the affected condyle and in non-affected condyle, the percentage difference and the right and left contribution were reported in Table 1.

**Table 1:** 18-F Fluoride PET/CT UCH patient results

Injected activity (MBq)	UCH patient affected side (R=right, L=left)	Increased uptake side (R=right, L=left)	SUVmax % difference	Right SUVmax	Left SUVmax	Right contribution	Left contribution
187	L	L	10,8%	11,5	14,3	44,6%	55,4%
190	R	R	11,8%	10,0	7,9	55,9%	44,1%
185	L	L	5,8%	14,7	16,5	47,1%	52,9%
187	L	L	3,6%	11,8	12,7	48,2%	51,8%
185	L	L	2,0%	10,1	10,5	49,0%	51,0%
185	R	R	1,4%	10,5	10,2	50,7%	49,3%
185	L	L	35,6%	19,5	41,1	32,2%	67,8%
189	L	R	4,4%	9,7	8,9	52,2%	47,8%
189	R	R	26,0%	16,2	9,5	63,0%	37,0%
190	L	L	9,6%	9,8	11,9	45,2%	54,8%
190	R	L	1,0%	9,3	9,5	49,5%	50,5%
195	L	L	10,0%	9,9	12,1	45,0%	55,0%
216	R	L	5,6%	9,3	10,4	47,2%	52,8%
221	R	L	1,5%	15,9	16,4	49,25%	50,75%
201	L	-	-	6,3	-	-	-
344	L	L	41,8%	7,3	17,8	29,1%	70,9%
370	R	None	0,0%	10,0	10,0	50,0%	50,0%
370	L	L	18,0%	10,9	15,7	41,0%	59,0%
370	R	R	15,4%	12,3	9,0	57,7%	42,3%
398	L	L	0,4%	12,6	12,7	49,8%	50,2%

Fifteen out of 19 patients showed a higher radiotracer uptake on the affected condyle suspected of active hypercondylia: 7 of them presented percentage difference > 10% and classified as suspected for active disease, while the other 8 patients presented a percentage difference < 10% and classified as suspected for inactive disease.

The median percentage difference of the group of patients suspected for active disease was 18 ± 12.2%, while it was 2.85 ± 3.42% for patient suspected for inactive disease.

Only 4 of 19 showed a lower uptake on the condyle suspected of UCH in comparison to the normal one. However, in all of these cases, the absolute value of the percentage difference was < 10% (respectively 1, 1.5, 4.3 and 5.6%).

Furthermore 2 of 19 patients underwent previously a Bone SPECT: 18F–NaF PET/CT and Bone SPECT showed, in both examinations, a significant increased radionuclide uptake on affected condyle. PET/CT and Bone SPECT were in agreement with each other and with the clinical suspicious.

Based on ICRP tabulated values the effective dose of a PET scan performed with 2.9 MBq/Kg of 18F–NaF (low administered activity) in a patient of 70 Kg, was about 3.5 mSv, while it was about 6.3 mSv for scan performed with 5.3 MBq/Kg (ordinary administered activity). The effective dose of a 99mTc-diphosphonates bone SPECT is 3.2 mSv in where a fixed value of 740 MBq of activity is injected. The effective dose to patients from CT scan was 0.15 mSv.

## Discussion

**Fig. 4:**
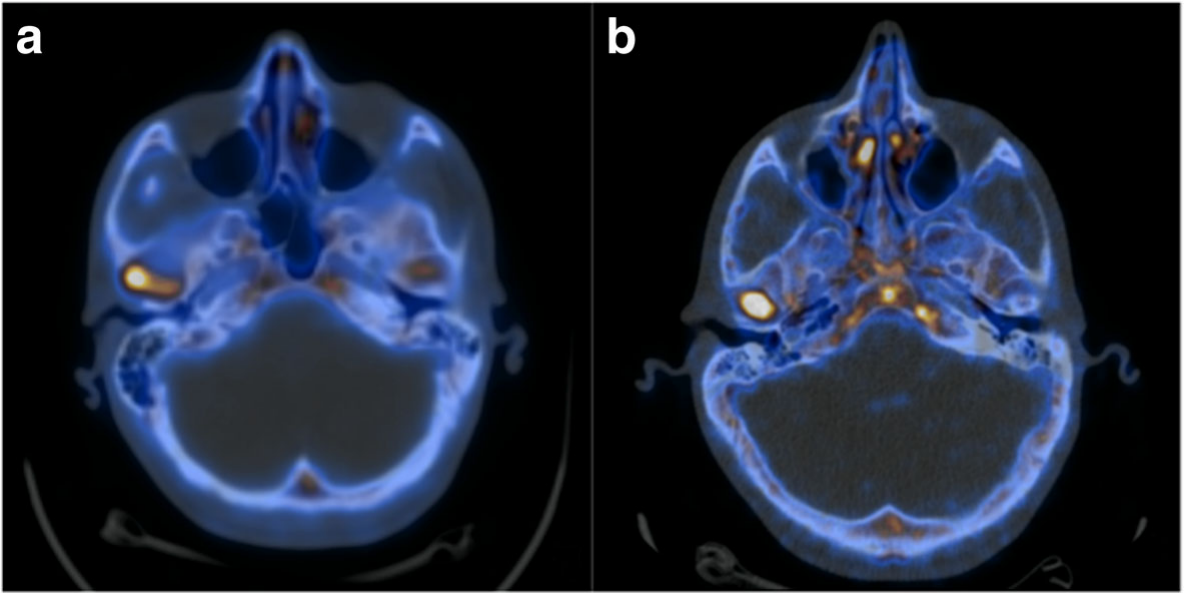
**a** PET/CT scan performed with an ordinary radiotracer activity [5.3 MBq/Kg of 18F–NaF]; **b** PET/CT scan performed with a low radiotracer activity [2.9 MBq/Kg of 18F–NaF]

18F–NaF PET/CT performed with a low radiotracer activity allows a good assessment of patient with UCH exactly as much as PET/CT performed with ad ordinary activity (Fig. 4).

Fluoride PET/CT shows good performance in condylar activity evaluation and it offers a valid pre-surgical guide to maxillofacial surgeon.

The effective radiation dose of a low-dose PET/CT is significantly lower than a standard-dose PET/CT (3.5 mSv vs 6.3 mSv).

The effective radiation dose of a low-dose PET/CT is not significantly higher than the most used Bone SPECT (3.5 mSv vs 3.2 mSv). Moreover PET/CT is performed in 1.5 h while Bone SPECT requires at least 3.5 h.

## Conclusions

PET/CT with 18F–NaF allows a good evaluation of the active or inactive state of growth of the mandibular condyle.

Based on our preliminary results, 18F–NaF PET/CT procedure could be performed, in UCH assessment, with 2.9 MBq/Kg of 18F–NaF (minimum 185 MBq, recommended 200 MBq) to minimize the effective radiation dose received by young patients without causing a significant deterioration of image quality.

The effective radiation dose of a low-dose PET/CT is not significantly higher than Bone SPECT and it ensures a better spatial resolution. However, the main concern of 18F–NaF PET/CT, if a cyclotron is not locally installed, is the cost of the radiotracer that is considerably higher than diphosphonates.

PET/CT could be performed in all of cases in which bone SPECT is not conclusive.

Further studies including a larger number of patients and clinical follow up are needed to confirm our preliminary findings.
